# The role of atomic fluorescence spectrometry in the automatic environmental monitoring of trace element analysis

**DOI:** 10.1155/S1463924693000136

**Published:** 1993

**Authors:** P. B. Stockwell, W. T. Corns

**Affiliations:** P S Analytical Ltd Arthur House B4 Chaucer Business Park Watery Lane, Kemsing, Kent Sevenoaks TN15 6QY UK

## Abstract

Considerable attention has been drawn to the environmental levels of mercury, arsenic, selenium and antimony in the last decade. Legislative and environmental pressure has forced levels to be lowered and this has created an additional burden for analytical chemists. Not only does an analysis have to reach lower detection levels, but it also has to be seen to be correct. Atomic fluorescence detection, especially when coupled to vapour generation techniques, offers both sensitivity and specificity.

Developments in the design of specified atomic fluorescence detectors for mercury, for the hydride-forming elements and also for cadmium, are described in this paper. Each of these systems is capable of analysing samples in the part per trillion (ppt) range reliably and economically. Several analytical applications are described.


Journal of Automatic Chemistry, Vol. 15, No. 3 (May-June 1993), pp. 79-84

### The role of atomic fluorescence spectrometry in the automatic environmental monitoring of trace element analysis

**P. B. Stockwell and W. T. Corns**

*P S Analytical Ltd, Arthur House, B4 Chaucer Business Park, Watery Lane, Kemsing, Sevenoaks, Kent TN15 6QY, UK*

*Considerable attention has been drawn to the environmental levels of mercury, arsenic, selenium and antimony in the last decade. Legislative and environmental pressure has forced levels to be lowered and this has created an additional burden for analytical chemists. Not only does an analysis have to reach lower detection levels, but it also has to be seen to be correct. Atomic fluorescence detection, especially when coupled to vapour generation techniques, offers both sensitivity and specificity.*

*Developments in the design of specified atomic fluorescence detectors for mercury, for the hydride-forming elements and also for cadmium, are described in this paper. Each of these systems is capable of analysing samples in the part per trillion (ppt) range reliably and economically. Several analytical applications are described.*

### Introduction

There is considerable concern about pollution in the environment; the focus of much of this has been on such heavy metals as mercury, arsenic, selenium, antimony and cadmium. A brief résumé of why some of these elements are important and where they are used is given below.

### *Mercury (Hg)*

The toxicological effect of mercury compounds on both plant and animal life has long been recognized, but it was not until the disaster at Minamata Bay in 1953 that the subject received worldwide attention [1]. Mercury occurs naturally in the environment in the form of mineral deposits and also anthropogenically from industrial and agricultural wastes. Because of its high toxicity there has been extensive research and development into techniques which can be used to determine mercury in a variety of samples.

In addition to the toxic nature of mercury, it is often present in natural gas and petroleum products, each of which can form the basis of feedstock for industrial reactions. Many of these take place in the presence of aluminium rotors or condensers. Even low levels of mercury can attack aluminium components, causing stress fractures [2]. These may result in a plant having to shut down for repairs, at a cost of millions of pounds.

### *Arsenic (As)*

In nature, arsenic seldom occurs in the free state, but, rather, is associated primarily with sulphide minerals of the general formula $X_mAs_nS_p$ where X = silver, copper or lead. All the inorganic and organic compounds of arsenic in the environment are in the $3^-$, $3^+$ or $5^+$ oxidation states.

Arsenic, and its various inorganic and organic compounds, figure in many industrial processes and applications, for example the manufacture of certain glasses, semiconductor and photo-conductor materials, lead and copper alloys, linoleum oil cloth, pigments, textile printing, antifouling paints, tanning, taxidermy, adhesives for metals, cotton desiccation, wood preservatives, herbicides, fungicides, rodenticides and insecticides. Such varied uses, coupled with the mining and smelting of arsenic-containing ores, coal combustion, waste incineration and agricultural burning, have resulted in a global atmospheric emission of 8 and 24 M kg/year from natural and anthropogenic sources, respectively. For many years there has been environmental concern regarding the possible contamination by arsenic of water, soil and food.

### *Selenium (Se)*

Selenium is of great significance in environmental analysis, biochemistry and medical chemistry. On the grounds of its ambivalent character (toxic, as well as essential), there is a particularly urgent need for an exact knowledge of the selenium content of biological and environmental materials. This is why there is a requirement for methods permitting analytical access to selenium concentrations ranging between increased (toxic) via normal to low (deficiency) levels. The element itself makes this task very difficult because the difference between increased and normal concentration levels is small, being only about one order of magnitude. Procedures with a high power of detection are required, especially where selenium is to be determined in its special binding forms, or when different species (Se [II], Se [0], Se [IV], Se [VI]) have to be detected, for example in water analysis.

### *Antimony (Sb)*

Although antimony is non-essential for life, it is found in biological specimens from people who have been exposed to industrial sources of antimony or who are being treated with drugs containing the element. Antimony is used in industry as a bearing metal, either by itself or alloyed

0142-0453/93 $10.00 © 1993 Taylor & Francis Ltd.

with tin and lead, and antimonial lead is used in storage batteries. Antimony compounds are used as flame retardants in plastics, polymers and rubber, and as pigments in paints. Other uses are in the manufacture of enamels, glasses and glazes and as mordants for textiles.

The main medicinal use of antimony compounds is in the treatment of parasitic diseases. In this context it is reported that pentavalent (V) antimony is less toxic than trivalent (III) antimony. Trivalent antimony is taken up by the red blood cells in humans, whereas pentavalent antimony remains in the plasma and it is more easily excreted than trivalent antimony.

The most likely route into the body from industrial exposure is by inhalation. This can arise from breathing the dust or fumes from the grinding and high temperature processing of antimony and its compounds or from stibine (antimony hydride) which is particularly poisonous.

*Cadmium (Cd)*

Cadmium is toxic to humans, it accumulates in the kidneys and too high a level results in renal failure. Unlike copper and nickel, it is *not* phytotoxic and it is readily taken up into plants, especially leaf vegetables. So if it is disposed to land it can result in human toxicity, whereas excess copper, nickel or zinc in a soil will probably kill the plants well before the humans can consume it.

Cadmium metal is very volatile compared with many other toxic metals and this is another major area of risk. Therefore if cadmium alloys are heated, there is a very serious risk due to the resulting cadmium vapour. The EC limit for drinking water is 5 μg/l. In the UK, the maximum permitted level is 3 mg/kg Ds for soils and also for sludge to land.

Cigarettes contain elevated levels of cadmium and raised Cd levels are detectable in the kidneys of smokers.

### Measurement

The elements discussed above are of environmental concern and therefore it is necessary to measure them accurately and reliably. Unfortunately, they are often present in small quantities; and they are difficult to analyse by conventional techniques.

Of the trace elemental analysis techniques, atomic fluorescence offers improved selectivity compared with optical emission spectrometry, because the resolution is determined by the line width rather than by the detection system. Fluorescence techniques offer good advantages in terms of linearity and detection levels. By its inherent nature the signal obtained can be increased by the intensity of the lamp or excitation source. The limitations are scatter and background levels of impurities. Atomic absorption, on the other hand, although it has been extensively used, suffers from the fact that it is non-linear and measurements at lower levels are extremely difficult. As legislative and environmental pressures increase, detection levels are required to be lower and lower. To maximize the analytical precision it is also important that the measurements, and therefore the sample pretreatment, are optimized to ensure reliable measurements. This often leads to single element analytical systems. Analysts are faced with the dilemma of achieving the detection levels promoted by instrument manufacturers. When the analyst is faced with the analysis of soil, biological samples and the like, the limit of detection required stretches analytical techniques. Fluorescence techniques often offer two orders of magnitide sensitivity improvement over absorption techniques and allow analysts to achieve the detection limited required and traceability of the results. Furthermore, in atomic absorption, non-specific absorption from molecular species may occur, resulting in positive bias. This phenomenon is not usually observed using fluorescence techniques [3].

### Theoretical aspects of atomic fluorescence

A typical atomic fluorescence arrangement, shown in figure 1, consists of an intense excitation source focused onto an atomic population in a flame. Fluorescence radiation, which is emitted in all directions, then passes to a detector in the same plane, usually positioned at right-angles to the incident light.

The source may be either an atomic line source or a continuum, and serves to excite atoms by the absorption of radiation at specific wavelengths. The atomics are then deactivated partly by collisional quenching with flame gas molecules and partly by emission of fluorescent radiation in all directions.

The wavelength of the fluorescence radiations is generally the same or longer than the incident radiation. The wavelengths of the emitted radiation is characteristic of the absorbing atoms and the intensity of the emission may be used as a measure of their concentration.

At low concentrations, this intensity is governed by the following relationship:

$$I_f = K\varnothing I_o C$$

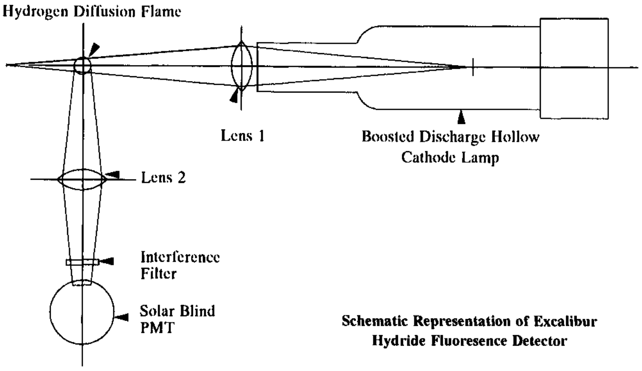

*Figure 1. Typical atomic fluorescence optical arrangement.*

where

$I_f$ = the intensity of fluorescent radiation.

C = the concentration of metal ion in solution.

K = a constant.

$I_o$ = the intensity of the source at the absorption line wavelength.

Ø = the quantum efficiency for the fluorescent process, which may be defined as the ratio of the number of atoms which fluoresce from the excited state to the number of the atoms which undergo excitation to the same excited state from the ground state in unit time.

There are five basic types of fluorescence which occur in flame measurement: (*a*) resonance fluorescence; (*b*) direct-line fluorescence; (*c*) stepwise-line fluorescence; (*d*) thermally assisted direct-line fluorescence; (*e*) thermally assisted anti-Stokes fluorescence. These have been described in detail by Thompson and Reynolds [4].

### Maximizing the benefits of atomic fluorescence

It is puzzling, considering its inherent advantages, that atomic fluorescence has not been more successful. Primarily this has been due to the matrix interference effects which occur when real samples are analysed. Coupling a fluorescence measurement technique to a vapour generation technique has the potential to overcome all of these problems with an additional bonus. The pretreatment to generate the vapour will remove a great majority of the interferents and the bonus is the increased transfer of efficiency of the element of interest to the measurement cell. Stockwell [5] has illustrated the improvements made by a vapour generation technique to the sensitivity of an ICP-OES system, i.e. by a factor of 500 times.

Each of the elements discussed here can be determined by vapour generation technique and each has accessible fluorescence lines, good available light source and therefore provide good prospects of measurement. The vapour generation techniques required involve: (*a*) generation of mercury itself as a vapour; (*b*) generation of the hydrides of As, Se, Sb, Te; and (*c*) generation of the tetraethyl cadmium species. P S Analytical, in association with Yorkshire Water Laboratory Services and the University of Plymouth, has pioneered the development of these coupled techniques and several instruments are now readily available for routine use.

### Determination of mercury by atomic fluorescence

Mercury is an ideal element for determination by fluorescence: it is atomic at room temperature and also absorbs and fluoresces at the same wavelength. Intense mercury sources are readily available and there is little problem in producing an atomic cell when the technique is coupled to vapour generation. Thompson and Godden [6] proposed the use of fluorescence for the determination of mercury in 1975. Godden and Stockwell [7] have described the development of a specific fluorescence spectrometer for mercury analysis, and this work resulted in a commercial instrument: the Merlin Fluorescence Detector.

The determination of mercury at low levels requires considerable care and attention to detail. Appropriate methodologies are described in Yorkshire Water's *Methods of Analysis* [8]. Basically the method requires the reaction of the samples in an acid medium with tin (II) chloride. This procedure is automated using a vapour generator, in which the sample, blanks and the tin (II) chloride are introduced using peristaltic pumps into a gas/liquid separator. Figure 2 shows a schematic representation of an automated instrument for mercury determination. The mercury is sparged from the gas/liquid separator by a flow of argon gas and the stream containing the mercury is fed into the Merlin Fluorescence Detector using a specific interface for analysis. The instrument measures total inorganic mercury and samples which contain organic mercury must be digested

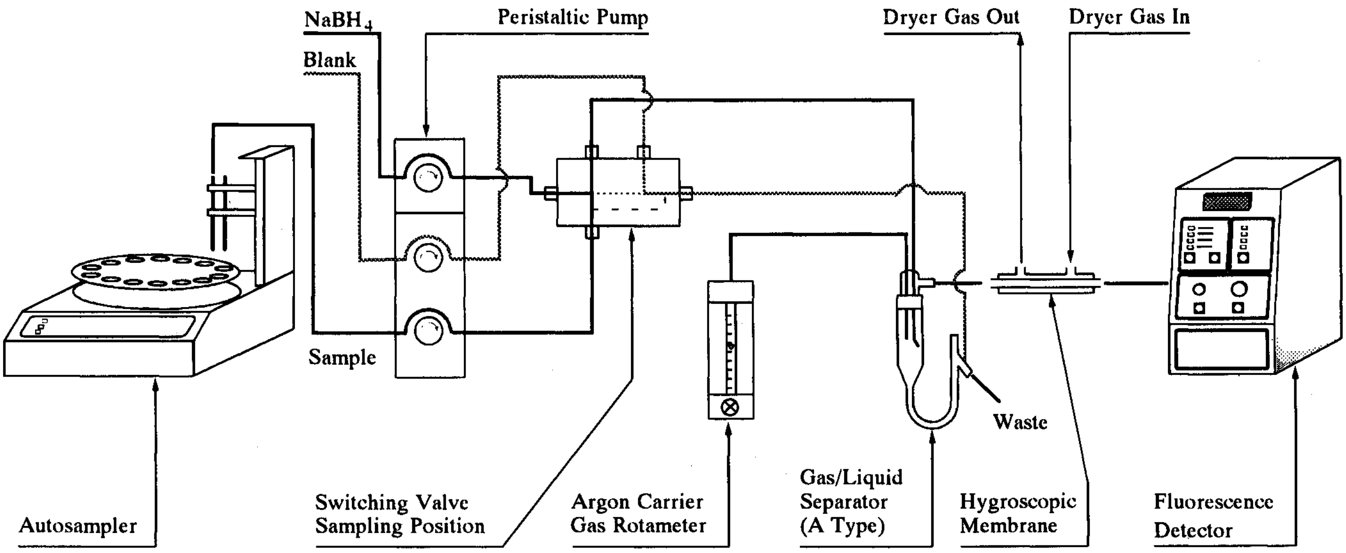

*Figure 2. Schematic representation of an automated system for mercury determination.*

prior to analysis. The preferred bromate/bromide digestion method ensures that complete breakdown of organomercury compounds is achieved within an hour [8]. Reagents and standards are prepared according to Yorkshire Water's *Methods*. The analytical instrument is set up according to the manufacturer's recommendations and allowed to stabilize. A quick check with coloured solutions will ensure that the equipment is functioning correctly. The reductant, reagent blank and samples are then presented to the vapour generator. The equipment is then calibrated over the range of interest. A typical signal response is shown in figure 3. Where the blank and sample matrices are completely matched, the resultant signal represents the true level of mercury in the sample. This signal represents the output typically obtained from 0·010 ppb of mercury in water. The calibration graphs are computed by the method of least squares and show excellent correlations which confirm the linearity and sensitivity of the method and Merlin's applicability to mercury analysis. The Merlin system provides a rapid, sensitive and fully automatic system for mercury determinations at ultra-trace levels. It can be applied to a wide range of samples, and systems are being successfully used in laboratory and process control applications. The accuracy of the technique is shown in table 1. Its performance exceeds all other commercial systems, even those relying on some method of preconcentration on gold traps etc.

### Determination of arsenic, selenium and antimony

Mercury is a relatively simple element to determine by fluorescence; however, the technique can be extended to other elements. Recent research by the authors with the University of Plymouth has looked at the science involved, as well as instrument optimization and simplification. The instrumentation concepts have focused on four main areas: (*a*) the light source; (*b*) the atom cell; (*c*) the optical design; and (*d*) read-out and data-processing.

For the determination of arsenic and other hydride forming elements, boosted discharge hollow cathode lamps have been found suitable for the excitation source of novel detectors. A simple hydrogen diffusion flame provides a viable atom cell.

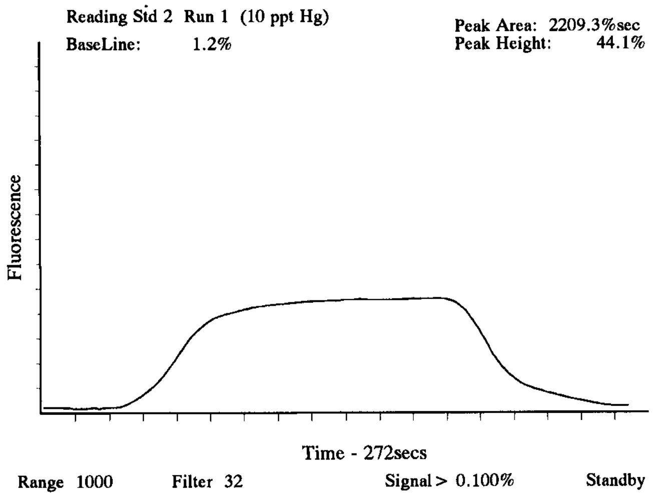

*Figure 3. Typical signal response for 0·01 ppb mercury using a continuous flow vapour-generator coupled to atomic fluorescence spectrometry.*

The optical design is more complex and requires attention to the selection of optical filters and reduction of scatter. Changing a lamp allows a range of detectors to be available commercially with little additional modification to the design. Data-processing and control facilities are provided using similar software as used with the Merlin Detector. Instrumentation for arsenic atomic fluorescence consists of a boosted discharge hollow cathode lamp as an excitation source, a hydrogen diffusion flame as an atom cell, a collection of lenses to collect and focus useful radiation, an interference filter to achieve wavelength isolation and a solar blind photomultiplier tube. Illumination from the excitation source is at right angles to the detection axis to suppress detection of radiation from the excitation source. A multi-reflectance filter specifically developed to maximize the optical transmission at the wavelength of interest has proved to be a versatile and sensitive option which allows measurement of arsenic, selenium, antimony and tellurium at significantly lower levels than can be achieved elsewhere [9]. Figure 4 shows the detectors for both mercury and the hydride forming elements. Both use similar data collection and output electronics.

The analytical operation of the arsenic fluorescence detection system is similar to that for mercury, but uses a different chemistry. Sodium tetrahydroborate is used instead of tin (II) chloride and its reaction with hydrochloric acid produces a steady stream of hydrogen.

*Table 1. Certified reference materials for mercury.*

| Certified reference material | Certified value | Value obtained |
|---|---|---|
| IAEA/W-4 Simulated water | 2.50 ± 0·13 μg/l | 2·45 ± 0·04 μg/l |
| Seronorm urine Batch 108 | 51·0 ± 5·0 μg/l | 59·0 ± 6·0 μg/l |
| BCR 143 Sewage sludge Amended soil | 3·92 ± 0·23 μg/g | 3·82 ± 0·13 μg/g |
| BCR 144 Sewage sludge Amended Soil | 1·39 ± 0·17 μg/g | 1·49 ± 0·22 μg/g |
| NIST SRM 16416 Fresh water | 1·52 ± 0·04 μg/ml | 1·47 ± 0·04 μg/ml |
| NIST RM 8407 River sediment | 50 ± 2 μg/g | 50 ± 1 μg/g |
| NIST RM 8408 River sediment | 107 ±2 μg/g | 106 ± 2 μg/g |
| Coniston Oil | 100 mg/l | 101 mg/l |
| BEST 1 Marine sediment | 0·092 μg/g | 0·094 ± 0·002 μg/g |
| NIST SRM 2670 Urine | 0·105 ± 0·008 μg/ml | 0·103 ± 0·005 μg/ml |

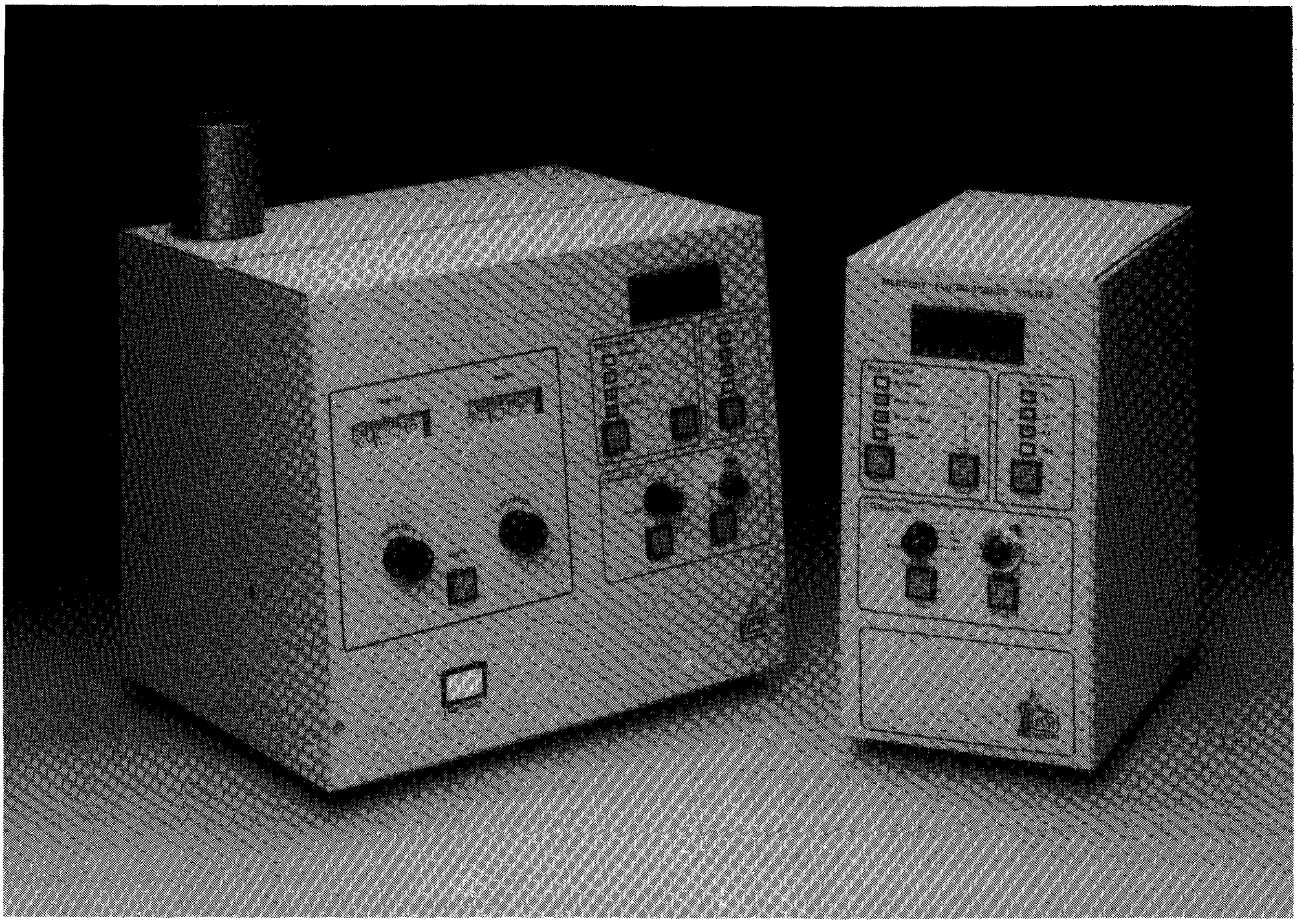

*Figure 4. Detectors for mercury and hydride forming elements.*

The hydrogen converts the arsenic (III) to the hydride form and provides the fuel for the atom cell (i.e. hydrogen flame). Arsine, produced by the reaction of the tetrahydroborate with the acid, is transferred by the argon stream into the hydrogen diffusion flame. The signal shape is similar to that produced for mercury, except that the plateau is achieved somewhat quicker and the memory effects are also minimized. Figure 5 shows a typical peak shape for 0·020 ppb arsenic solution.

The calibration graph is linear between 0–100 ppb of arsenic. Typically, detection levels in the region of 0.010 ppb are achievable using the vapour generator coupled to the specific fluorescence detection system described above. The accuracy of the technique is shown in table 2, where the results for certified water reference materials are given. Selenium, antimony and tellurium are also determined in a similar fashion.

### Determination of cadmium as tetraethyl compound

With the success of vapour generation approaches for mercury and the hydride forming elements, the extension to other elements, especially those with good atomization sources such as cadmium and lead is obvious. Several groups [10, 11] have investigated the conversion of cadmium and lead to the tetraethyl compounds. Goodall and co-workers [12] coupled the PSA vapour generation technology to a specific atomic fluorescence detector for cadmium determination. The technology is similar to that used for the Excalibur detector, except that no hydrogen is produced by the chemical reaction to form the tetraethyl compound. An additional flow of hydrogen/argon is required to create the atomization cell. In

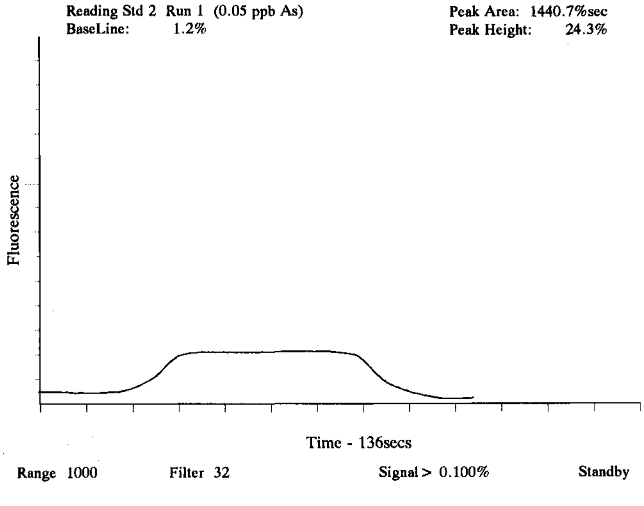

*Figure 5. Typical signal response for 0·02 ppb arsenic using continuous flow hydride generation coupled to atomic fluorescence spectrometry (Excalibur, PSA).*

*Table 2. Certified reference materials: arsenic.*

| Certified reference material | Certified value (μg/l) | Value obtained (μg/l) |
|---|---|---|
| CASS2 (Near-shore seawater) | 1·01 ± 0·07 | 1·08 ± 0·18 |
| SLEW1 (Estuarine water) | 0·765 ± 0·093 | 0·697 ± 0·020 |

addition, a cadmium discharge lamp is used as the atomization source, rather than a boosted hollow cathode lamp – and the power supply module is simpler in concept. The optics and data collection systems are basically those designed for the Excalibur. A prototype instrument has been constructed along the above lines and initial results draw significant promise. Detection levels in the low ppt levels are possible and the performance for certified reference materials are shown in table 3.

### Conclusion

Atomic fluorescence is already proving to be a very reliable and sensitive means of determining mercury, arsenic, selenium and antimony. Recent research has clearly demonstrated that it is also useful for the measurement of cadmium and lead. Commercially available detectors are likely to be available by the end of 1994.

*Table 3. Certified reference materials: cadmium.*

| Certified reference material | Certified value | Value obtained |
|---|---|---|
| NIST 1643c (Water) | 12·2 ± 0·1 μg/l | 12·6 ± 0·5 μg/l |
| BCR 144 (Sewage sludge) | 3·41 ± 0·25 μg/g | 3·34 ± 0·15 μg/g |
| BCR 145 (Sewage sludge) | 18·0 ± 1·2 μg/g | 18·24 ± 0·7 μg/g |

### Acknowledgements

The advancements in the analytical applications of atomic fluorescence could not have been achieved so successfully and in a such a short time scale without assistance from a number of sources. Their assistance is gratefully acknowledged here. Professor L. Ebdon, Dr P. Goodall and Dr S. Hill from the University of Plymouth are thanked, as is Dr Clive Thompson of Yorkshire Water Lab Services, Sheffield. The methods for mercury in water were developed at Yorkshire Water Lab Services, Sheffield Laboratory, Charlotte Rd, Sheffield S2 4EQ, UK.

### References

1. Smith, W. E. and Smith, A. E. (Eds), *Minimata.* Holt, Rinehardt and Winston, New York, 1975.
2. English, J. J., A mercury induced aluminium alloy piping failure in an ethylene manufacturing plant. Paper presented at the First Ethylene Producers Conference, 4 April 1989. American Institute of Chemical Engineers.
3. West, C. D., *Analytical Chemistry*, **46** (1974), 797.
4. Thompson, K. C. and Reynolds, R. J., *Atomic Absorption, Fluorescence and Flame Emission Spectroscopy, A Practical Approach.* Charles Griffin & Co., 1978.
5. Stockwell, P. B., *Laboratory Practice*, **39** (1990), 29.
6. Thompson, K. C. and Godden, R. G., *Analyst*, **100** (1975), 544.
7. Godden, R. G. and Stockwell, P. B., *Journal of Analytical Atomic Spectrometry*, **4** (1989), 301.
8. Yorkshire Water, *Methods of Analysis*, 5th edition. Yorkshire Water, Leeds, 1989 (Section 9).
9. Corns, W. T., Stockwell, P. B., Ebdon, L. C. and Hill, S. J., *Journal of Analytical Atomic Spectrometry*, **8** (1993), 71.
10. D'Ulivo, A. and Chen, Y., *Journal of Analytical Atomic Spectrometry*, **4** (1989), 319.
11. Sturgeon, R. E., Willie, S. N. and Berman, S. S., *Analytical Chemistry*, **61** (1989), 1867.
12. Goodall, P., Hill, S. J., Ebdon, L. C., Stockwell, P. B. and Thompson, K. C., *Journal of Analytical Atomic Spectrometry* (in press).

